# Type IX collagen gene mutations can result in multiple epiphyseal dysplasia that is associated with osteochondritis dissecans and a mild myopathy

**DOI:** 10.1002/ajmg.a.33240

**Published:** 2012-03-26

**Authors:** Gail C Jackson, Dominique Marcus-Soekarman, Irene Stolte-Dijkstra, Aad Verrips, Jacqueline A Taylor, Michael D Briggs

**Affiliations:** 1 Wellcome Trust Centre for Cell Matrix Research, Faculty of Life Sciences, University of ManchesterManchester, UK; 2 Regional Molecular Genetics Service, St. Mary's HospitalManchester, UK; 3 Department of Clinical and Cytogenetics, University HospitalMaastricht, the Netherlands; 4 Section Clinical Genetics, Department of Genetics, University Medical Center GroningenGroningen, the Netherlands; 5 Neuromyologist Department of Child Neurology, Canisius-Wilhelmina HospitalNijmegen, the Netherlands

**Keywords:** multiple epiphyseal dysplasia, myopthathy, type IX collagen, cartilage, osteochondritis dissecans

## Abstract

Multiple epiphyseal dysplasia (MED) is a clinically variable and genetically heterogeneous disease that is characterized by mild short stature and early onset osteoarthritis. Autosomal dominant forms are caused by mutations in the genes that encode type IX collagen, cartilage oligomeric matrix protein, and matrilin-3: *COL9A1*, *COL9A2*, *COL9A3*, *COMP*, and *MATN3*, respectively. Splicing mutations have been identified in all three genes encoding type IX collagen and are restricted to specific exons encoding an equivalent region of the COL3 domain in all three α(IX) chains. MED has been associated with mild myopathy in some families, in particular one family with a *COL9A3* mutation and two families with C-terminal COMP mutations. In this study we have identified *COL9A2* mutations in two families with MED that also have osteochondritis dissecans and mild myopathy. This study therefore extends the range of gene-mutations that can cause MED-related myopathy. © 2010 Wiley-Liss, Inc.

## INTRODUCTION

Multiple epiphyseal dysplasia (MED) is a clinically and genetically heterogeneous disease that manifests with joint pain and stiffness, mild short stature, and degenerative joint disease [Fairbank, 1947; Barrie et al., 1958; Rimoin et al., 1994]. In addition, several phenotypically overlapping disorders have been shown to be allelic with the more classical forms of MED, including bilateral hereditary micro-epiphyseal dysplasia (BHMED) [Mostert et al., 2002]. The identification of the causative gene mutations has allowed the classification of MED based on molecular genetic criteria as well as clinical and radiographic features. It has become clear that there is considerable allelic and non-allelic genetic heterogeneity within MED, which is reflected in the extensive clinical and radiographic variability of the MED phenotype. Mutations identified in patients with MED have now been found in the genes encoding cartilage oligomeric matrix protein (*COMP*), matrilin-3 (*MATN3*), and type IX collagen (*COL9A1*, *COL9A2*, and *COL9A3*) for the autosomal dominant forms, while mutations in *SLC26A2* cause autosomal recessive MED [Briggs and Chapman, 2002].

Several reports have described families with MED that was associated with other complications such as osteochondritis dissecans (i.e., the delamination of articular cartilage from the underlying subchondral bone) [Versteylen et al., 1988] and mild myopathy or muscle weakness [Bonnemann et al., 2000; Jakkula et al., 2003; Kennedy et al., 2005b]. More recently a large family with MED, which was caused by a mutation in *COL9A3*, presented with muscle weakness and a muscle biopsy showed mild myopathy that was characterized by a variability in fiber size [Bonnemann et al., 2000].

We had the opportunity to study two large families with MED that was complicated by osteochondritis dissecans and myopathy. We evaluated the clinical and molecular attributes of these disorders in the two families to better understand the relationship of these rare manifestations.

## PATIENTS AND METHODS

### Family 1

**Fig 1:**
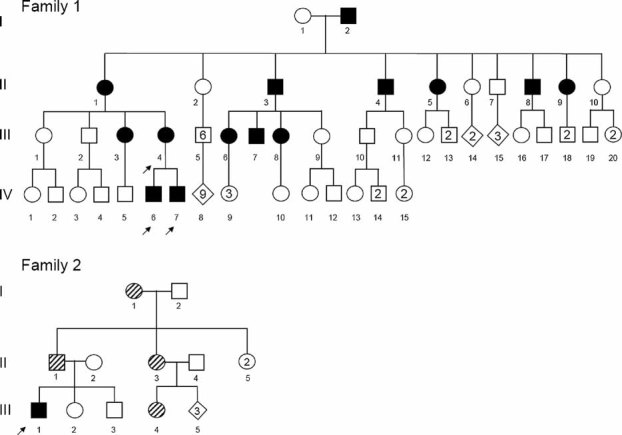
Pedigrees of two the families with MED presented in this study. An arrow indicates those individuals in which a *COL9A2* mutation was identified. In Family 2 those individuals with semi-filled symbols were reported as affected by II-1, but this has not been independently confirmed by clinical or radiographic examination.

**Fig 2:**
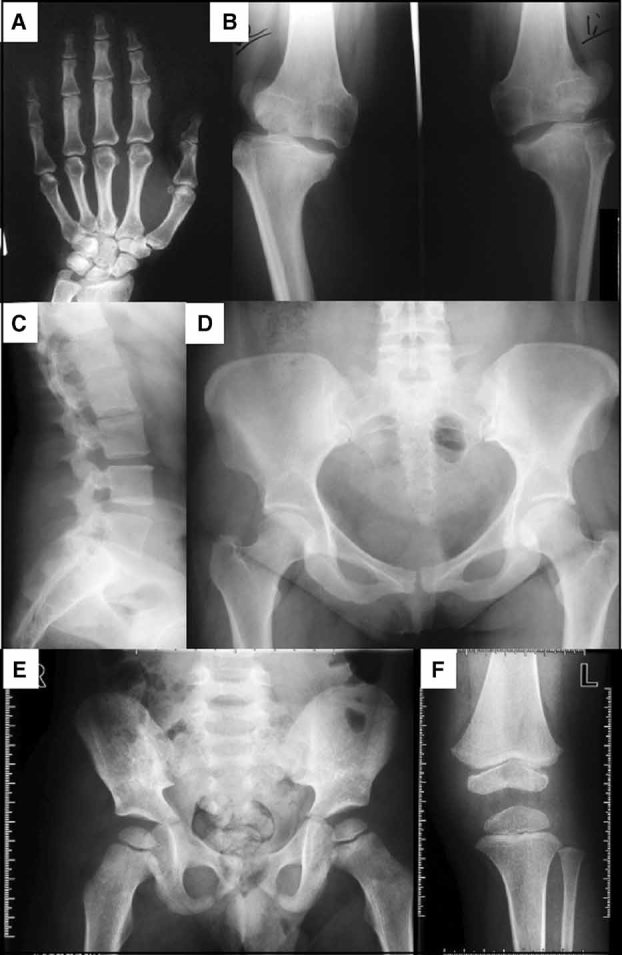
Radiographs of the proband (individual III-4, panels A–D) in Family 1 at 33 years of age and her eldest son (individual IV-6, panels E,F) at 3 years of age. The mother's radiographs showed (A) normal hand, (B) knees with bilateral *genu varus*, irregular joint surfaces, and decreased joint space, especially of the right knee, (C) lateral spine with irregular endplates of the vertebral bodies in the thoracolumbal region and (D) relatively normal hips. Radiographs of her 3-year-old son showing (E) hips and (F) the left knee. The radiographs were consistent with a diagnosis of mild MED, characterized by delayed ossification of the epiphyses. The hips were relatively spared but had small proximal femoral epiphyses, while the knees had small femoral and tibial epiphyses.

This family has been reported previously [Versteylen et al., 1988], an update and additional clinical and molecular data are reported here. A 33-year-old woman visited out-patient clinic suffering from stiff and painful knees and hands, which had started from the age of 4 years [Patient B3 in Versteylen et al., 1988 and individual III-4 in Fig. 1A]. At that time her height was 1.54 m (−2.5 SD), arm span 1.53 m, weight 84 kg (>2 SD), OFC 56 cm (0 to +1 SD) and ICD 3.5 cm (+2 SD). Radiographs of the knees were available and showed Blount's disease (progressive varus deformity of the proximal tibia associated with internal torsion of the tibia) and imaging of the spine showed mild spondyloarthrotic abnormalities in the thoracic region (Fig. 2). The patient's family had been reported in the past to have osteochondritis dissecans of the knees as part of a phenotype that overlapped with MED and was inherited in an autosomal dominant fashion. Her sister (individual III-2) had suffered painful and swollen knees from the age of 4 years and later in life she also developed severe osteochondritis dissecans [Patient B4 in Versteylen et al., 1988 and individual III-3 in Fig. 1A]. This patient was not re-evaluated in this current study.

The eldest son of the proband (individual IV-6) was developing similar problems as the rest of his family and at the age of 4 years he started to complain of pain in the knees during walking. His height at age 5 years was 1.15 m (0 SD), arm span 1.09 m, weight 18.5 kg (−2 to 0 SD), OFC 53.5 cm (+1 SD), ICD 3.2 cm (+1 to +2 SD), and OCD 8.3 cm (−1 SD). He had frontal bossing and a depressed nasal bridge. The mother reported that he could not run like his classmates and that he was easily fatigued. An examination by a child neurologist showed weakness of the abdominal musculature. For example, moving from a supine position to an upright sit took a long time, but was possible without the use of his arms. There were no other abnormalities except for a positive Trendelenburg sign on both sides of the hip. A muscle biopsy taken at the age of 5 years showed some variation in fiber size, but was otherwise normal. Radiographs of his knees and hips were consistent with a mild form of MED (Fig. 2).

The second son (individual IV-7) was born with a birth weight of 4.510 kg (large for gestational age) after a normal pregnancy. By the age of 4 years he was also developing similar complaints as his brother, most notably pain in the knees, clumsy walking, tiredness, and easy exhaustion. He showed the same facial features as his brother. His height was 1.02 m (−1 SD), arm span 99 cm, weight 16.9 kg (0 SD), OFC 52.5 cm (0 to +1 SD), ICD 3 cm (+1 SD), and OCD 9 cm (0 to +1 SD). Radiographs of the knees at that age showed similar abnormalities to his brother (data not shown).

### Family 2

**Fig 3:**
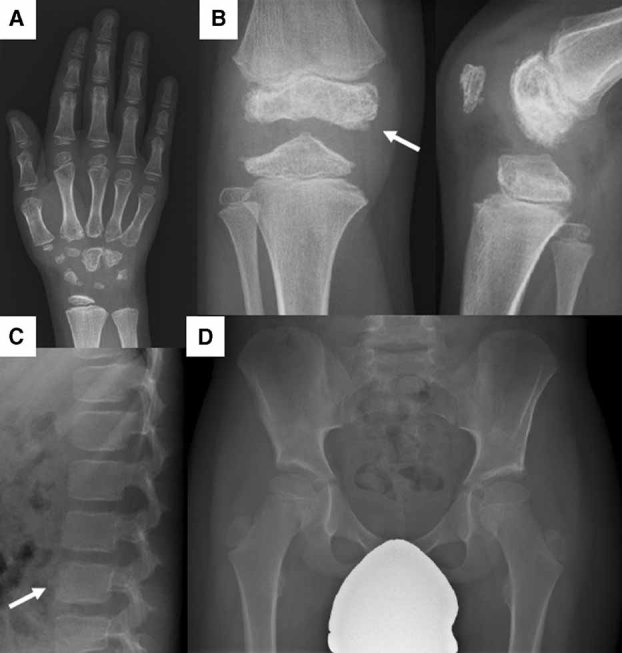
Radiographs of the proband in Family 2 (individual III-1) at age 9 years and 10 months of age showing (**A**) hand, (**B**) knee, (**C**) spine, and (**D**) hips. The radiographs were consistent with a diagnosis of MED, characterized by delayed and abnormal endochondral ossification. The hand showed marked carpal ossification delay with irregular carpal bones with a coarse internal structure. The distal epiphysis of the radius was also irregular and small. The knees had small and irregular femoral and tibial epiphyses and irregular metaphyses. The pelvis showed some flattening of the proximal femoral epiphyses but they were relatively spared. There was some irregularity of the vertebral end plates (arrow).

The index patient (individual III-1, Fig. 1B) was referred to the Department of Clinical Genetics after it was noted on a radiograph that his carpals had an abnormal shape (Fig. 3). He had been seen at the age of 3 years because of an abnormal gait. Neurological evaluation at that time showed normal development, with normal reflexes, normal response to sensation and normal muscle tone, but he had a wide based gait and an inability to lift his feet off the ground when running. In fact he sometimes used a Gowers' maneuver to get up. Creatine kinase and lactate levels were normal. A left sided developmental dysplasia of the hip was diagnosed, which was surgically corrected. His gait however remained stiff and he had long-term physiotherapy to improve his motor skills. At the age of 9 years he had mild knee and foot pain after prolonged walking. His height was 140.8 cm (0 SD), weight 33.1 kg (0 SD), sitting height 75.7 cm (0 to +1 SD), arm span 138.9 cm (O SD), and head circumference 54.8 cm (+1 SD). He had a slightly flat midface and there was one tooth missing in the lower jaw. Ophthalmologic examination was normal. His younger brother and sister (individuals III-2 and III-3) did not have similar complaints. The family history was positive for osteochondritis dissecans and hypodontia. The father had osteochondritis dissecans of the right elbow during puberty, but at the time of this report he was almost symptom free with occasional mild complaints of knee pain. He also had four missing teeth. His height was 190.8 cm (+1 SD), arm span 181.0 cm (−1 SD), and a sitting height of 105.2 cm (+2 SD). One of his sisters (individual II-3) was wheel chair bound for a year during puberty and she was reported to have had severe osteochondritis dissecans of the knees, elbows, and sternocostal joints. After puberty her complaints subsided and at the time of this report she was fully mobile but there was some muscle weakness. Two of her children (individuals III-4 and III-5) were also reported as affected and one of her affected children has some teeth missing. The paternal grandmother was noted to have asymptomatic osteochondritis dissecans on radiographs taken when she 60 years of age (data not shown). Only individuals II-1, II-2, III-1, III-2, and III-3 were seen by a clinical geneticist (I-SD) since the rest of the family are currently residing abroad.

### Mutation Analysis

Samples of DNA from affected members of both families were collected for mutation analysis in the genes known to cause MED. Screening of the *COMP* and *MATN3* genes was undertaken as described previously [Jackson et al., 2004; Kennedy et al., 2005a; Zankl et al., 2007]. Briefly, exons 8 to 19 of *COMP* and exon 2 of *MATN3* were amplified by PCR and subject to bi-directional DNA sequencing, but this analysis did not reveal any mutations. We therefore screened for mutations in exon 3 of *COL9A2* and *COL9A3* and exon 8 of *COL9A1* (NM_001851, NM_001852, and NM_001853, respectively) and the immediate splice donor and acceptor sites. Briefly, PCR amplifications (Primer Sequences; COL9A1Ex8F 5′-GGCCTCCTCTGGAAGGTAA-3′, COL9A1EX8R 5′-AGTCCTGCCCTTTCCTATTCT-3, COL9A2EX3F 5′-TAGGGGACCTGGACAGAAGA-3′, COL9A2EX3R 5′-TCCCTTGAAAACAGAGATGGA-3′, COL9A3EX3F 5′-GTTCTTGAGGGACCCCTGA-3′, COL9A3EX3R 5′-AATGACCCCTCTGTTCTGAG-3′) were performed in a total volume of 20 µl using a custom PCR ReadyMix™ master mix (ABgene, Epsom, UK) containing 0.5 µM of each primer, 1.25 U of *Taq* polymerase, 67 mM Tris–HCl (pH 8.0 at 25°C), 16 mM (NH_4_)_2_SO_4_, 3.7 mM MgCl_2_, 0.085 mg/ml BSA, 6.7 mM EDTA, 0.75 mM of each dNTP and 50–100 ng of genomic DNA. Thermal cycling for PCR consisted of an initial denaturation at 95°C for 3 min, followed by 30 cycles of denaturation at 95°C for 30 sec, annealing at 62°C for 30 sec, and extension at 72°C for 1 min, followed by a final extension at 72°C for 3 min. Purified PCR products were used as a template for bi-directional fluorescent DNA sequencing and the sequence data were compared against a reference trace and a negative control sequence to detect variants using the Staden sequence analysis package [Staden et al., 2000].

### Results of Genetic Analysis of Type IX Collagen Genes

Affected members of Family 1 (the proband and both of her affected sons) were heterozygous for c.186+2T>C in the splice donor sequence of intron 3 of *COL9A2*. The index patient of Family 2 was heterozygous for c.186G>A in the splice donor sequence of exon 3. Nucleotide numbering is according to mRNA sequence with Genebank accession number for *COL9A2* = NM_001852 and nucleotide 1 has been counted as the first nucleotide of the translation initiation codon.

Both of these mutations have been identified in families with MED and shown to result in the skipping of exon 3 sequence from *COL9A2* mRNA [Muragaki et al., 1996; Holden et al., 1999].

### Histological Analysis of a Muscle Biopsy

Microscopic examination (hematoxylin and eosin staining) of a muscle biopsy from the *m. vastus lateralis* obtained at the age of 5 years from the eldest son of the proband (individual IV-6, Family 1) showed no significant morphological or histochemical abnormalities (see supporting information Fig. 1 which may be found in the online version of this article). There was no evidence of muscle fibers with central nuclei (i.e., indicative of fiber stress and remodeling); however, some variation in fiber size was observed (5–35 µm with a mean diameter of 24 µm), consistent with previous data [Bonnemann et al., 2000]. No degeneration, regeneration, or necrosis was present and there was a normal fiber type distribution (ATPase staining). The periodic acid-Schiff, myophosphorylase, naphtyl-esterase, NADH, SDH, and COX stains were all normal. To evaluate the exercise-induced leg pain and fatigue, his mitochondrial respiratory chain substrate oxidation rates, ATP metabolism, and enzyme activities were measured in the biopsy. The ATP + CrP production from pyruvate was diminished (22.3; normal range 42.1–81.2 nmol/hr mUCS), [1 -^14^C]pyruvate and malate was 3.15 nmolCO_2_/hr/UCS (normal range 3.61–7.48), [1,4 -^14^C]succinate and acetylcarnitine was 2.08 nmolCO_2_/hr/UCS (normal range 2.54–6.39). The other substrate oxidation rates and the enzyme activities (complex I–V, citrate synthase) were normal.

## DISCUSSION

In this study we identified mutations in the splice donor site of exon 3 in *COL9A2* that results in MED in two unrelated families. These data are consistent with previous studies showing that MED mutations in *COL9A2* reside exclusively in the splice donor sequence of exon 3 [Muragaki et al., 1996; Holden et al., 1999; Spayde et al., 2000; Fiedler et al., 2002; Takahashi et al., 2006]. The strict grouping of MED mutations in the splice donor and acceptor sites of exon 3 of *COL9A2* and *COL9A3*, respectively, and the splice acceptor site of exon 8 of *COL9A1*, is yet to be fully explained.

The association of myopathy with MED has been noted before and is associated with mutations in both *COL9A3* [Bonnemann et al., 2000; Lohiniva et al., 2000] and *COMP*, in particular mutations in those exons of *COMP* that encode the C-terminal domain of COMP [Jakkula et al., 2003; Kennedy et al., 2005b]. However, myopathy does not appear to be a consistent feature of MED caused by *COL9A3* mutations [Paassilta et al., 1999; Nakashima et al., 2005] and it has been suggested that the degree of muscle weakness correlates with the severity of the MED phenotype [Nakashima et al., 2005]. The results of the present study show that mutations in *COL9A2* can also cause myopathy and suggests an important role for type IX collagen in the musculoskeletal system. Furthermore, like *COL9A3* mutations, myopathy is not a consistent feature of MED caused by *COL9A2* mutations. Indeed, unrelated MED families with the same mutations identified in this study do not present with muscle weakness [Muragaki et al., 1996; Holden et al., 1999]. It is becoming increasingly clear that there is considerable inter- and intra-familial variability in the phenotypic severity of MED caused by type IX collagen gene mutations [Paassilta et al., 1999; Bonnemann et al., 2000; Lohiniva et al., 2000; Nakashima et al., 2005]. This is a similar finding to that seen in MED caused by *MATN3* mutations [Jackson et al., 2004; Makitie et al., 2004] and it is interesting to speculate that the genetic modifiers of phenotypic severity may be common across the spectrum of the pseudoachondroplasia-MED (PSACH-MED) bone dysplasia family, regardless of which gene the causative mutation resides in.

The muscular component of the MED phenotype in these patients is poorly understood, but has been characterized by either increased protein kinase C levels or atrophic fibers observed in muscle samples obtained through biopsy. In the patient reported here (individual IV-6 in Family 1) and also the patient described in a previous report [Bonnemann et al., 2000], the muscle at the site of biopsy showed only mild variations in fiber size, even though there were clinical symptoms of a mild myopathy in both families. The Trendelenburg sign, as noted in the patient reported here, reflects a weakness of the abductor muscles of the hip. If this is indeed the case then it is possible that the muscle biopsy was obtained from the wrong region (i.e., from the *m. vastus lateralis* instead of the *gluteus medius* and *minimus muscles*), or alternatively the Trendelenburg sign is a secondary consequence of either a shortening of the femoral neck or a painful hip dysplasia. A recently described mouse model of mild PSACH-MED caused by a C-terminal COMP mutation (p.Thr585Met) [Pirog-Garcia et al., 2007] also showed signs of myopathy that was characterized by fibrosis, necrosis, and variable fiber diameter in the muscle. However, these pathological changes were restricted to the myotendinous junction, where COMP and type IX collagen are expressed, and suggested that the observed myopathy may be a secondary consequence of an underlying tendinopathy [Piróg et al., 2010]. It is therefore possible that the muscle weakness seen with type IX collagen gene mutations may also arise from a tendinopathy and detrimental changes in the forces conveyed during locomotion.

The clinical biochemistry of the eldest affected son of Family 1 (individual IV-6) showed diminished ATP and CrP production and mildly reduced pyruvate, malate, succinate, and acetylcarnitine levels in muscle. These are all markers of the oxidative capacity of mitochondria and the decreased levels in the muscle biopsy suggest mitochondrial dysfunction in the patient. There is an increasing body of data that demonstrates that endoplasmic reticulum (ER) or cell stress can directly trigger mitochondrial cell death pathways [Guicciardi and Gores 2008]. For example, Hori et al. 2002 have shown that the suppression of protein synthesis, due to ER stress, has a detrimental effect on the synthesis of mitochondrial-associated proteins. These can include the cytochrome oxidase (COX) subunits and ATP-dependent proteases and/or chaperone proteins contributing to assembly of the COX complex. Recent studies have shown that the expression of mutant *COMP* and *MATN3* mutations can cause ER stress [reviewed in, Bateman et al., 2009], the consequences of which have been reduced chondrocyte proliferation and increased apoptosis in vivo. This detrimental change in chondrocyte phenotype is independent of whether the mutant protein is retained in the rough ER [Leighton et al., 2007] or efficiently secreted [Pirog-Garcia et al., 2007]. We hypothesize that the expression of mutant type IX collagen may elicit a cell stress response that has down stream consequences with respects to cell and mitochondrial function and viability. Type IX collagen is not expressed in skeletal muscle [Irwin et al., 1985; Muller-Glauser et al., 1986], however, it is present in the fibrocartilagenous tissue at the bone-tendon interface and it is possible that the expression of mutant type IX collagen in this tissue might cause an underlying tendinopathy that ultimately affects a wide range of musculoskeletal tissues. This hypothesis is consistent with the myopathy in a mouse model of mild PSACH-MED resulting from a *Comp* mutation [Piróg et al., 2010].

Further analysis of patients and mice models of PSACH-MED will help clarify the pathology and disease mechanisms of mild myopathy resulting from type IX collagen and COMP mutations.

## Notes

Fig 1 Microscopic examination (H & E staining) of a muscle biopsy at the age of 5 years from the m. vastus lateralis of individual IV-6 (family 1) showed no significant morphological or histochemical abnormalities. Some variation in fiber size was observed (5–35·m with a mean diameter of 24·m), but there was no evidence of central nuclei (arrow heads), which is considered a marker of muscle stress and remodeling.
