# Supplementary figures and images for: Type IX collagen gene mutations can result in multiple epiphyseal dysplasia that is associated with osteochondritis dissecans and a mild myopathy
Source: Am J Med Genet A. 2012 Mar 26;152A(4):863–9. doi: 10.1002/ajmg.a.33240 (PMC3557369; doi:10.1002/ajmg.a.33240)

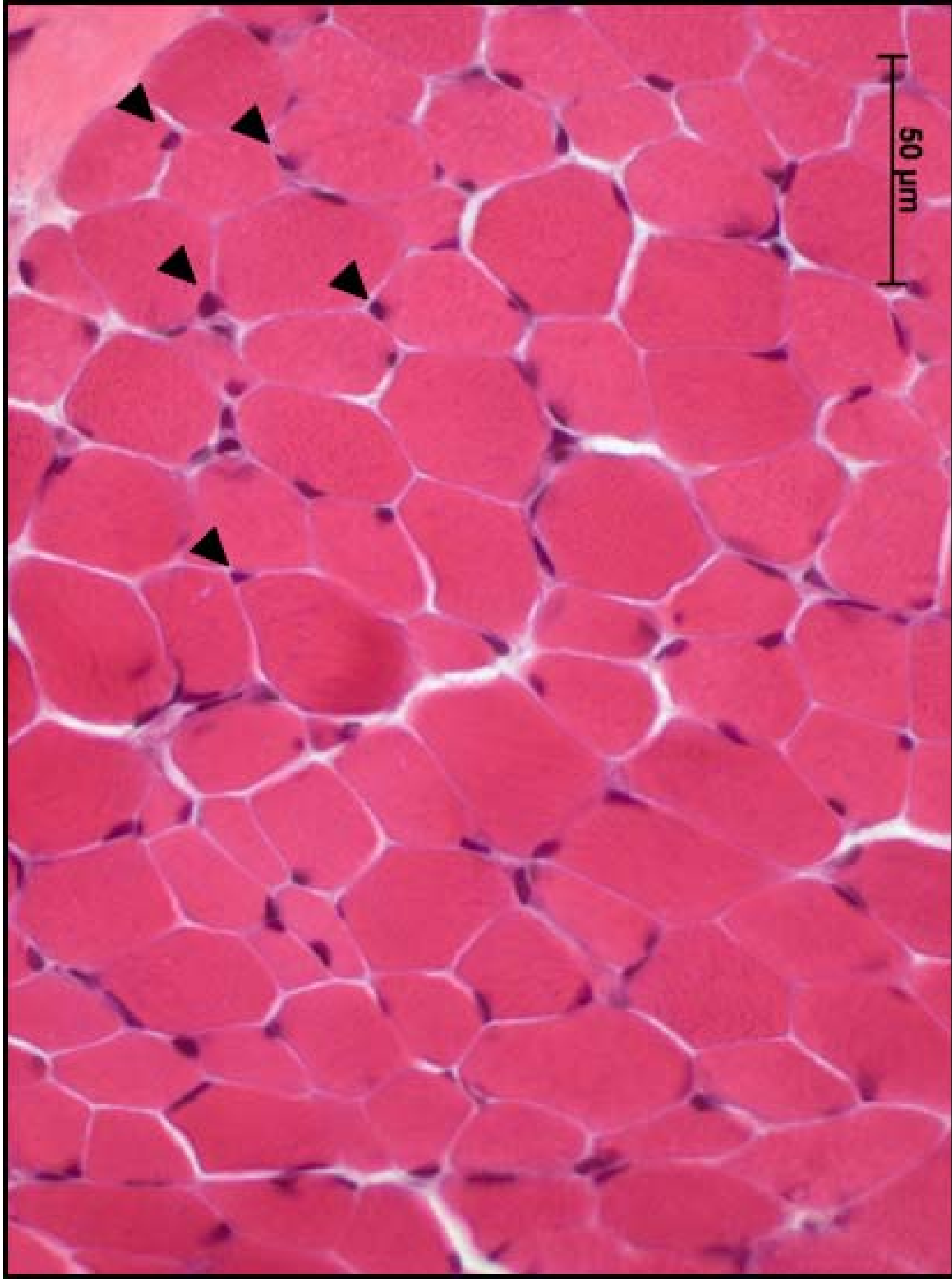

Supplement: Fig 1 — Microscopic examination (H & E staining) of a muscle biopsy at the age of 5 years from the m. vastus lateralis of individual IV-6 (family 1) showed no significant morphological or histochemical abnormalities. Some variation in fiber size was observed (5–35·m with a mean diameter of 24·m), but there was no evidence of central nuclei (arrow heads), which is considered a marker of muscle stress and remodeling. [file ajmg0152-0863-SD1.tif]
